# The cancer stem cell: Evidence for its origin as an injured autoreactive T Cell

**DOI:** 10.1186/1476-4598-5-6

**Published:** 2006-02-14

**Authors:** Peter Grandics

**Affiliations:** 1 A-D Research Foundation 5922 Farnsworth Ct, Carlsbad, CA 92008, USA

## Abstract

This review explores similarities between lymphocytes and cancer cells, and proposes a new model for the genesis of human cancer. We suggest that the development of cancer requires infection(s) during which antigenic determinants from pathogens mimicking self-antigens are co-presented to the immune system, leading to breaking T cell tolerance. Some level of autoimmunity is normal and necessary for effective pathogen eradication. However, autoreactive T cells must be eliminated by apoptosis when the immune response is terminated. Apoptosis can be deficient in the event of a weakened immune system, the causes of which are multifactorial. Some autoreactive T cells suffer genomic damage in this process, but manage to survive. The resulting cancer stem cell still retains some functions of an inflammatory T cell, so it seeks out sites of inflammation inside the body. Due to its defective constitutive production of inflammatory cytokines and other growth factors, a stroma is built at the site of inflammation similar to the temporary stroma built during wound healing. The cancer cells grow inside this stroma, forming a tumor that provides their vascular supply and protects them from cellular immune response.

As cancer stem cells have plasticity comparable to normal stem cells, interactions with surrounding normal tissues cause them to give rise to all the various types of cancers, resembling differentiated tissue types. Metastases form at an advanced stage of the disease, with the proliferation of sites of inflammation inside the body following a similar mechanism. Immunosuppressive cancer therapies inadvertently re-invigorate pathogenic microorganisms and parasitic infections common to cancer, leading to a vicious circle of infection, autoimmunity and malignancy that ultimately dooms cancer patients. Based on this new understanding, we recommend a systemic approach to the development of cancer therapies that supports rather than antagonizes the immune system.

## Introduction

Understanding the pathomechanism of cancer is of primary interest in medical research. In the past century, several mechanisms were proposed: It was hypothesized that cancer arises out from a single cell that loses its differentiated state through sequential mutations [1]. This initiation-promotion-progression concept explains the steps in a sequential process [2]. Later, this hypothesis led to the mutagenic and recently the oncogenic theories which hypothesize that defects in tumor suppressor genes are responsible for the development of cancer [3]. The impairment of cell-to-cell communication as a cause of cancer has also been postulated [4].

Mutations and other genetic abnormalities observed in cancer cells could also be caused by environmental effects, e.g., chemical carcinogens or life style factors such as alcohol or tobacco consumption or drug abuse [5]. The discovery of the cancer stem cell [6-8] lent support to the theory that cancer may develop out of a single cell, and raised the question of cancer stem cells arising from normal stem cells [9]. Indeed, if normal stem cells could undergo the type of mutations observed in tumor cells, this would potentially compromise the genetic stability of the organism. Therefore, the likelihood that normal stem cells are extremely well protected is demonstrated by their resistance to radiation and toxins [9].

One fascinating finding is that immunosuppressive cytotoxic antineoplastic therapies may on occasion cause the regression of a clinically established cancer. At first, applying this as a therapeutic strategy may seem counterintuitive, considering the fundamental role of the immune system in protecting the body against infectious organisms and aberrant cells. In addition, cancer itself is frequently immunosuppressive, so exacerbating a pre-existing immunosuppression may not seem like a rational strategy.

In this light, it appears paradoxical that the same degree of immunosuppression that is lethal in a bacterial or fungal infection actually benefits cancer suppression. In other words, the deletion of the T cell compartment that accompanies cytotoxic antineoplastic therapies [10] may facilitate cancer regression. This suggests that cancer itself may arise out of the immune system, potentially from the T cell compartment, which would explain why the suppression of cellular immunity could also lead to the suppression of the disease.

Another observation is that tumor cells are poorly immunogenic, despite the fact that tumor cells are antigenic [11,12]. Therefore, they do not generate a T cell-mediated immune response, and if so, it is of low intensity [13]. If tumor cells were derived from injured lymphocytes, particularly T cells that still share some functional properties with their normal counterparts, an immune tolerance to cancer cells could be explained, as the immune system is not made to attack itself. In pathological situations, T cells do attack self-tissue in a manner reminiscent of the autoreactive nature of cancer cells which have the ability to attack and invade host tissues. In other words, cancer cells behave like autoreactive lymphocytes. Here, we explore the evidence suggesting that such a mechanism could be at work during the development of cancer.

The prevalent genetic theories of cancer are built upon observations of genetic abnormalities in tumor cells. These theories do not generally take into account the demonstrated importance of environmental factors in human cancer development. In a previous paper [14] we have shown that specific dietary deficiencies mimic the effects of chemical or radiation damage to DNA, which we propose plays an important role in human carcinogenesis and tumorigenesis. This observation allows us to consider cancer as a single disease, possibly developing from a single cancer stem cell. Based on this, we could assume that the observed genomic abnormalities in cancer cells are an effect rather than the cause of the disease. This idea also points to the direction of upstream events preceding the development of the malignant cell. We propose that identifying these events will be fundamental to understanding the pathomechanism of cancer. By exploring the functional similarities between lymphocytes and cancer cells, we provide an insight into this realm of possible upstream events.

### The exterior cell surface layer (cell coat)

The lymphocyte cell coat is a labile structure, and the treatment of cells may lead to the loss of its components [15-20]. The cell coat plays an important role in lymphocyte functions including homing, cell mediated immunity, electrophoretic properties and antigen expression [21]; cell surface proteins are thought to be involved in cell propagation and differentiation [18]. After treatment with β-glucosidase [22], sialidase [23,24] and trypsin [25], lymphocytes lose their homing abilities. Cytotoxic lymphocytes transiently lose their cytotoxic ability after a brief papain treatment [26]. Lysis of the cell coat suppresses cell-mediated immunity [27-29]. Treatment by glycosidases including neuraminidase affects the bodily distribution of lymphocytes [23,24] and demonstrates alterations in their antigenicity [30-34]. Treatment with trypsin and neuraminidase reversibly eliminates the mitogenic response of lymphocytes [35,36]. The cell coat on thymocytes is significantly thicker than on splenic lymphocytes, [20] suggesting a role for the cell coat in T cell function. The cell coat of the lymphocyte cell membrane has been characterized using various stains [15-17], [37-39]. These investigations found high acid mucopolysaccharide content with a significant number of acidic amino sugar end groups.

Cancer cells also exhibit an exterior cell surface coat [40-45]. The similarities between the cell coat of normal and leukemic lymphocytes have been investigated [39,41]. Pathological lymphocytes (CLL) have a uniformity of staining similar to their normal counterparts, with some differences observed with cationic stains that could be due to a decrease in the sialoprotein of the cell coat of CLL cells. With some similarity to lymphocytes, the tumor cell coat has been suggested to play a role in cell contact and adhesion, cell recognition [44], as well as the capacity to metastasize [46].

The tumor cell coat is also sensitive to neuraminidase [47-49] and can rapidly re-grow following treatment with the enzyme [50]. The enzyme treatment also changes the immunological properties of tumor cells. Trypsin and EDTA removes the tumor cell coat [51]. The cell coat is involved in the mechanism by which tumor cells escape cellular immune attack [45,52-54]. The degradation of the cell coat by brief hyaluronidase treatment of glioma cells sensitizes them to cytotoxic lymphocyte attack [52,53]. Although normal human glial cells also produce hyaluronic acid, glioma lines produced significantly more. Hyaluronidase-sensitive coats have been found on a variety of murine sarcoma and carcinoma cell lines [54]. It appears that a mucopolysaccharide coat on tumor cells impedes the successful use of immunotherapy. It was demonstrated that the displacement of the tumor cell coat by charge-functionalized lipids or polycationic substances leads to tumor cell apoptosis and tumor destruction [45,55,56].

It is demonstrated that the cell coat of lymphocytes and tumor cells are functionally significant. The degradation/removal of cell coat significantly impacts the functionality of both tumor cells and lymphocytes; therefore, tumor cell isolation methods could alter the functionality of isolated cells. In other words, with the loss of the cell coat, lymphocytes lose fundamental functions, i.e., cannot attack target cells, while tumor cells also lose cell contact and adhesive properties, as well as the ability to metastasize. In addition, tumor cells become sensitive to apoptosis.

### Activation of coagulation

The activation of coagulation occurs during tissue injury as well as in various pathologies. Infection leads to an inflammatory reaction as well as the activation of coagulation, as there is a crosstalk between these functions [57-59]. Blood coagulation components can inhibit or amplify the inflammatory response. Blood clotting is initiated when pathogenic components such as endotoxin or inflammatory cytokines induce the synthesis of tissue factor on leukocytes [60]. The coagulation cascade is subsequently triggered. The formation of negatively charged membrane phospholipid surfaces amplifies the coagulation reaction [61]. Natural anticoagulant pathways such as the protein C anticoagulant pathway limit the coagulation process, thereby suppressing the inflammatory response including reducing inflammatory cytokine secretion [62], decreasing NF-κB signaling [63], minimizing leukocyte chemotaxis [64] and endothelial cell interactions [65], and suppressing apoptosis [66].

Platelets are also involved in the link between inflammation and coagulation. Inflammatory cytokines such as IL-6 or IL-8 increase platelet production, and such platelets are more thrombogenic [67]. In addition, the platelets release the CD40L protein, a potent proinflammatory mediator, which subsequently induces tissue factor synthesis [68,69] and amplifies the secretion of proinflammatory cytokines [70,71]. This in turn leads to a progressive cycle that ultimately can produce severe vascular and organ injury.

In 1865, Trousseau first described a cancer-associated condition now called migratory thrombophlebitis in which a spontaneous coagulation of the blood occurs in the absence of inflammatory reactions [72]. It manifests as migratory thrombosis in the superficial veins of the chest wall and arms, but it can occur in other sites as well. This condition is a variant of venous thromboembolism. Thrombosis is a frequent complication of malignancy, and thromboembolic death is the second leading cause of mortality in cancer [73,74]. Malignant cells interact with the blood coagulation system by releasing procoagulant and fibrinolytic substances and inflammatory cytokines [75-85]. In addition, direct interaction with endothelial cells, monocytes/macrophages, and platelets also leads to localized clotting activation [85-87]. Similar to normal activated inflammatory cells, malignant cells release tissue factor [75-77] which promotes the formation of fibrin deposits in the tumor cell microenvironment [88-90].

The fibrin gel matrix along with other connective tissue components form the basis for the tumor stroma, a matrix in which tumor cells are dispersed and which provides the vascular supply as well as a barrier against rejection by the cellular immune system [89]. The tumor stroma shares properties in common with the temporary stroma of a healing wound [91]. Similar to the fibrin coating on macrophages [92], the observed fibrin coating of tumor cells is involved in the mechanism by which tumor cells escape destruction by NK cells [93,94]. Histological evidence suggests that inflammatory lymphocytes are confined to the tumor-host interface, and do no not significantly penetrate the tumor [89,95]. Malignant cells secrete inflammatory cytokines such as TNF-α and IL-1β that downregulate the anticoagulant system of vascular endothelial cells [96,97]. The secretion of IL-8 promotes new blood vessel formation, [98] and the fibrin deposited around tumor cells facilitates angiogenesis [99-101].

Tumor cells attach to the vascular endothelium and promote the adhesion of leukocytes and platelets [102-105]. Monocytes and macrophages also home in on vascular surfaces due to inflammatory stimuli [106-108]. In response to inflammatory molecules, complement, lymphokines and immune complexes, these cells subsequently secrete procoagulant tissue factor; tumor-associated macrophages express significantly higher levels of tissue factor than control cells [109,110]. These macrophages also increase their fibrinolytic enzyme production [111].

Both human and animal cancer causes platelet aggregation *in vitro *and *in vivo *[112-114]. The ability of tumor cells to aggregate platelets and secrete plasminogen activator correlates with their metastatic potential [115]. Indeed, thrombocytopenia reduces the metastases of tumors [116,117] as do compounds capable of reducing platelet aggregation [117-125]. These include aspirin, prostaglandins and other nonsteroidal (NSAID) anti-inflammatory drugs. A reduced risk of fatal colon cancer has been observed among aspirin users [120-122]. Administration of heparin and fibrinolysin also reduces the incidence of experimental metastases [126-128], while the administration of anti-fibrinolytic agents increases their incidence [129,130].

Cancer treatment by surgery, cytotoxic antineoplastic drugs and hormonal therapy all contribute to the hypercoagulable state and risk factors for thromboembolism in cancer patients [131,132]. The risk of fatal pulmonary embolism increases four-fold after surgery in cancer patients [133,134]. Chemotherapy drugs including cysplatin, mytomicin C and tamoxifen as well as high-dose and multi-drug regimes increase the risk of thrombotic complications [135-139]. Prophylactic treatment with warfarin reduces this risk (140). The use of hematopoietic growth factors subsequent to chemotherapy was shown to induce thrombosis in breast cancer patients [141,142]. Venous thrombosis could also be a marker for an otherwise asymptomatic cancer [143,144].

Similarly to a normal inflammatory reaction, activation of coagulation takes place in cancer. The events of tumor stroma development are comparable to wound healing [91] and it is possible that tumor formation may be associated with defective wound healing initiated by an inflammatory reaction due to infection and/or tissue injury. Therefore, we believe it is important to investigate potential links between infection, inflammation and cellular immune response in searching for the origins of the cancer cell.

### Infection and inflammation

The etiological role of infectious agents has been indicated in various cancers. In 100 cases of human leukemia, *Mycoplasma*, *Salmonella*, *Micropolyspora*, *Mycobacterium*, *Absidia*, pseudorabies virus and adenovirus antigens were commonly detected in the patient's sera [145]. Hepatotropic viruses (hepatitis B and C) cause hepatic necrosis followed by hepatocellular, B cell and gastric malignancies [146-149]. Antiviral therapy of hepatitis C infection led to the regression of virus-associated B cell lymphoma [150]. Adenoviral infection has been associated with childhood leukemia [151] and cytomegalovirus infection with testicular cancer [152]. *Helicobacter pylori *infection is widespread in the population (an estimated 40–80% infected) and is linked to gastric cancer and mucosa-associated lymphoid tissue (MALT) lymphoma [153,154]. A reversal of lymphoma-induced neutropenia has been observed with the eradication of *H. pylori *infection [154]. Simian virus 40 (SV40) is associated with human brain cancers and non-Hodgkin's lymphoma [155]. Ocular adnexal lymphoma is linked to *Chlamydia psittaci *infection, and the reversal of lymphoma was observed with pathogen-eradicating antibiotic therapy [156]. The list continues: Cervical intraepithelial neoplasia (CIN) is associated with human papilloma virus (HPV) infection with a co-etiological presence of chronic bacterial cervicitis [157-159]. *Mycoplasma *and HPV association was found to be dominating. The role of mycoplasma in the dysplasia of the uterine cervix and development of CIN has also been demonstrated [160].

Mycoplasmas are particularly interesting due to their widespread presence in the human population. Although many mycoplasmas are not directly pathogenic in humans, they are associated with many diseases [161-165]. Mycoplasmas have co-leukemogenic activity [166-168] and are found to increase tumor cell invasiveness [169]. In approximately half of the examined cases, mycoplasma DNA was present in ovarian and gastric carcinoma specimens [170,171]. In gastric, lung, esophageal, breast and colon cancers as well as glioma specimens, *Mycoplasma hyorhinis *was detected in about 50% of the cases [172]. Mycoplasmas are known to cause chromosomal changes [173]. Mixed *Mycoplasma pneumoniae *and influenza virus infection induced lung cancer in an animal model [174]. The direct role of the AIDS-associated *Mycoplasma fermentans *and *Mycoplasma penetrans *in oncogenesis has been investigated [175]. These mycoplasma strains induced gradual malignant transformations that eventually became irreversible. Besides its direct oncogenic potential, *Mycoplasma fermentans *was found to exhibit a unique cytocidal effect on the undifferentiated myelomonocytic lineage, but not on differentiated myelomonocytic cells [176]. The depletion of immature myelomonocytic cells likely contributes to the functional immunodeficiency present in cancer patients.

In response to pathogens, the host mounts a protective inflammatory response. Immune cells migrate to the area of infection and produce inflammatory messengers called cytokines. Initially, cells of the innate immune system (macrophages, neutrophils, NK cells) become involved, followed by the activation of cells of the adaptive immune system. These include antigen-presenting cells (APCs), T and B cells, which play an important role in propagating the inflammatory response. T cell inflammation plays a major role in antitumor immune responses. Key regulators of T cell-mediated response are the T helper (Th) cells that secrete the cytokines orchestrating this response. The two subtypes Th1 and Th2 cells produce cytokines stimulating cellular and humoral immune responses.

Intracellular pathogens (e.g., viruses, mycoplasmas) use the Toll-like receptor (TLR) signaling mechanism to escape host defenses [177]. Pathogen-associated molecular patterns on the surface of mycoplasmas engage TLRs 1, 2, and 6 on the surface of APCs that lead to a Th2-type polarization of the immune response and the secretion of IL-10, IL-4, IL-5 and IL-13 [178-180]. These cytokines are antagonistic to Th1 type cytokines (TNF-α, IL-2, IFN-γ, IL-6, IL-12); excessive production of either type of cytokine upsets the homeostatic balance needed to maintain a proper mix of cellular and humoral immune responses. Utilizing this mechanism, mycoplasmas suppress cell-mediated immunity, which allows them to persist and predispose the host for colonization by other pathogens. The observation that leukemia patients were colonized by over half a dozen pathogens besides mycoplasmas [145] suggests that suppression of the cellular immune system provides a fertile ground for a variety of pathologies.

Besides regulating innate and adaptive immune responses, cytokines are involved in cell growth and differentiation. Normally, the secretion of cytokines is of short radius and limited duration, typically regulating self or adjacent cell functions. The activity of cytokines is tightly regulated, and there is evidence that cytokines contribute to inflammatory autoimmune diseases [181-184] and malignancies. Similarly to activated T cells, various tumor cells secrete immune response-polarizing cytokines (IL-10, IL-6, IL-8, IL-13, TGF-β) serving as autocrine and/or paracrine growth factors for the cancer [185-199]. The progression of the disease and patient survival was correlated with increasing levels of cytokine secretion [200]. This secretion is frequently constitutive, leading to elevated serum levels of cytokines in malignancies including melanoma, non-small cell lung carcinoma, renal cell carcinoma and bladder carcinoma [186-190,201]. In addition, tumor cells can induce IL-10 in the tumor environment [191]. IL-10, the most potent Th2 polarizing cytokine, suppresses the tumoricidal activity of macrophages [202], blocks presentation of tumor antigens to professional APCs [203-205], and inhibits tumor-specific cytotoxic T cells [206]. However, in cancers both cellular and humoral immune response may be depressed, as in the absence of IL-4 production IL-10 secretion alone cannot induce a Th2-type response.

It appears that the immune response becomes distorted at multiple levels during the development of cancer. First, infectious agents may act in concert to subvert cellular immunity, thereby upsetting the homeostatic balance of a proper mix of cellular and humoral immune response. This leads to an aberrant cytokine-signaling that results in depressed apoptosis and excessive proliferation [207,208]. Cytokines seem to be the key substance of apoptosis of leukemic cells [207]. Abnormal inflammatory cytokine secretion by tumor cells reinforces the existing imbalances and thus promotes disease progression. Similarly to T cells, cancer cells use inflammatory cytokines as autocrine and paracrine growth factors, suggesting a functional relationship between cancer cells and cells of the immune system.

### Infection, autoimmunity and cancer

Several lines of evidence suggest a direct relationship between infection, autoimmunity and cancer. Hepatitis B and C viruses are involved in an autoimmune condition that precedes the development of hepatocellular carcinoma [209]. Data also demonstrate a higher prevalence of B-cell non-Hodgkin's lymphoma in HCV-infected patients with autoimmune manifestations [147-149] including Sjorgren syndrome [210], cryoglobulinemia [211,212] and systemic lupus erythematosus (SLE) [213,214]. Adenovirus infection is associated with childhood leukemia, (151) and family studies in acute childhood leukemia have shown possible associations with autoimmune disease [215]. Epstein-Barr virus [216] and human T lymphotropic virus type 1 infection [217] is associated with abnormal lymphoproliferation and Hodgkin's lymphoma. Cytomegalovirus infection is linked to autoimmunity [218] and testicular cancer [152].

*H. pylori *infection can lead to autoimmune neutropenia and MALT-lymphoma [154] in addition to its well-established role in the development of gastric cancer. Systemic rheumatic disease has also been linked to lymphoid malignancy [219]. These findings underline a close relationship between infection, autoimmunity and proliferative disorders, possibly mediated by an abnormally functioning cytokine signaling network [220].

Antinuclear antibodies (ANA) were demonstrated in the sera of 19% of patients with malignancies in the absence of overt autoimmune manifestations [221]. In cancer patients, a large number of autoantibodies are observed against tissue-specific antigens, nucleoproteins, membrane receptors, proliferation-associated antigens, tissue-restricted antigens, etc. [reviewed in [222]]. Autoimmune connective tissue disorders are also commonly associated with malignancies [223]. It was reported that gastric atrophy and pernicious anemia carries a risk for gastric carcinoma 18 times that of the population average [224]. It appears that a variety of infections may induce autoimmune serological features without overt autoimmune disease or organ involvement [225]; however, this condition may progress to clinical autoimmune disease and malignancy if impaired T cell function prevails. Such condition develops at a higher frequency among the elderly [226].

It was observed 30 years ago that a low percentage of human T cells (3.4%) have the ability to form auto-rosettes with autologous erythrocytes; in breast cancer and melanoma patients, the ratio was elevated to 6.1% and 7.4%, respectively [227]. This observation implied that some level of autoreactivity is normal, confirmed later by studies on T cell tolerance [228,229]. However, the observation also pointed to an elevated level of autoreactive T cells involved in cancer. The mechanism of activation of an autoreactive T cell response was linked subsequently to bacterial and viral infections through the process of molecular mimicry [218,230-234] in which pathogen-derived peptides mimic self-peptides. This phenomenon was studied in animal models [235-240] and was supported by clinical observations [241-243]. As a highlight, when lymphocytic choriomeningitis virus (LCV) antigens were expressed in the pancreas of transgenic mice, infection with the virus led to autoimmunity and diabetes [239].

*H. pylori *antigens mimic epitopes on H^+^, K^+^-adenosine triphosphatase in the gastric mucosa [230] thereby activating cross-reactive gastric T cells. Viral peptides mimic sequences on myelin basic protein [234], leading to multiple sclerosis. Cytochrome c (cyt c) as an antigen was used to study how self-proteins prime autoreactive T cell responses [244,245], as SLE patients possess autoantibodies to cyt c [246]. When non-self cyt c was co-administered with the self-protein, B cells specific for the foreign antigen primed autoreactive T cells that led to breaking tolerance to self-cyt c. The same autoimmune phenomenon occurs in the LCV transgenic mice when LCV antigens on pancreatic cells and the intact virus antigens are co-presented to the immune system [239]. Therefore, it is quite likely that autoimmunity spontaneously develops during a variety of infections when antigens on microorganisms mimic self antigens and are presented together, breaking T cell tolerance.

The presence of autoreactive T cells has been observed in healthy persons, which indicates a role for these cells in immune defense. If autoreactive T cells were always absent from the T cell repertoire, the responsiveness toward foreign antigens that resemble self-antigens would be reduced. This notion is supported by the observation that T cells which recognized variants of self-antigen are of lower avidity than those recognizing a foreign antigen [247,248]. Also, tolerance to self-antigen reduced T cell variants for these peptides as well as the diversity of T cell receptor α and β-chain sequences of self-specific T cells [249,250]. It appears that some level of autoreactive T cells is necessary for immune defenses. Clinical autoimmunity may develop when persistent infection provides a continuing high dose of antigenic stimulus, [251] and this situation could predispose patients for the development of proliferative disorders.

### Defective apoptosis

Normal tissue development requires damaged, dangerous or unnecessary cells to be eliminated while healthy cells survive. The survival of harmful or damaged cells can lead to various pathologies. The evolutionarily conserved mechanism of apoptosis eliminates unwanted or abnormal cell populations. Lymphocytes require IL-2, IL-4, IL-7, IL-9 and IL-15 for viability [252,253], and withdrawal of these cytokines leads to apoptotic cell death. Leukemia patients who went into complete remission following chemotherapy developed a different type of leukemia after being placed on IL-2 therapy [185]. IL-2 is an essential cytokine for the viability of activated T-cells [254], suggesting a link between the survival of activated T-cells and leukemic cells. Myeloid leukemia cells are also cytokine-dependent and undergo apoptotic cell death following cytokine withdrawal [253]. The various immune response-polarizing cytokines that tumor cells secrete [185-201] inhibit chemotherapy- or radiation-induced apoptosis [256-261]. There are myeloid leukemia cell lines that have become independent of an external cytokine supply [257], but generally cytokines can protect both normal and cancer cells against apoptosis induced by various cytotoxic agents. The persistence of infectious agents and chronic inflammation in cancer patients promotes NF-κB activation and inflammatory cytokine production, thereby contributing to the diminished apoptosis of abnormal cells [262,263].

The completion of immune response against pathogenic microorganisms requires the deletion of activated T and B cells that participated in the immune defenses, particularly self-reactive ones [264] (although a fraction of them survive as memory cells). Apoptosis plays an important role in the regulation of peripheral immunity through the Fas/APO-1 cytotoxic pathway. Defective apoptosis can lead to autoimmune disease [265,266] and cancer [267,268]. As cancer cells are not immortal, they maintain a program for apoptotic cell death [269].

The apoptosis marker Fas receptor (FasR) is expressed on numerous cell types, whereas the Fas ligand (FasL) is mainly expressed on T cells [266]. FasL mediates the apoptosis of effector T cells as part of an immune response termination and tolerance development. FasL is also expressed in "immune-privileged" tissues such as the brain, testes and eyes with the purpose of preventing inflammation. Mutations in Fas or FasL can lead to autoimmune disease [270,271]. Similarly to cytotoxic T cells, various tumor cells also express FasL and use it to induce apoptosis of invading lymphocytes. Breast tumor cells express FasL that can kill Fas-sensitive lymphoid cells [272]. The co-expression of Fas and FasL was observed in brain tumors that can use this mechanism to obtain a proliferating advantage by "counter-attacking" tumor-infiltrating activated Fas-sensitive T lymphocytes [273,274]. Similar observations have been made in Ewing sarcoma [275], gastric cancer [276], cholangiocarcinoma [277], B cell chronic lymphocytic leukemia (B-CLL) [278], colon adenocarcinoma [279-281], head and neck cancer [282], lung carcinoma [283], esophageal carcinoma [284], ovarian carcinoma [285], lymphoma [286], pancreatic carcinoma [287], melanoma [288], and other malignancies [289,290]. Childhood glial tumor cells (but not normal cells) in the brain express the common leukocyte-associated antigen and Fas [273].

The expression of apoptosis-related molecules on the surface of both neoplastic cells and cytotoxic lymphocytes (CTL) in tumor specimens raises the question of whether neoplastic cells are formed from CTLs by a premature termination of the apoptotic mechanism. Indeed, neoplastic cells behave like CTLs in their expression of FasL and in the induction of apoptotic death of activated T cells, as well as other cancer cells carrying a functional FasR [291,292]. In other words, cancer cells continue to act like T cells performing their immune-regulating functions.

## Discussion and therapeutic implications

Infections by various pathogenic microorganisms are a common occurrence in humans and other animals. In response to invading pathogen(s), an inflammatory reaction develops in the host organism. Initially, the innate immune system becomes involved, followed by the development of an adaptive immune response. Activated leukocytes produce inflammatory cytokines and chemokines as well as other growth factors aimed at clearing up the infection and facilitating tissue healing. The inflammatory reaction at the infection site triggers a variety of physiological responses. Antigen-presenting cells activate T and B cells in response to molecular patterns expressed on the surfaces of pathogenic microorganisms. Intracellular pathogens are overcome by the cellular immune response; in addition, the T cell inflammatory reaction is also key to antitumor immunity. Activated T helper 1 (Th1) cells secrete specific cytokines orchestrating this response.

Pathogenic microorganisms, however, have evolved strategies to evade immune surveillance in order to persist in the host. Several intracellular pathogens including mycoplasmas and viruses deploy molecular patterns on their surfaces that trigger a Th2-type (humoral) immune response and consequently depress cellular immunity. In addition, some infections such as the mycoplasmas remain sub-clinical, and by subverting the cellular immune response, these microorganisms predispose the host for colonization by other pathogens eventually leading to various pathologies.

Molecular mimicry is initiated when viruses integrate host genes within their genome, [293] and pathogens with host-like genes may have a survival advantage over those lacking such traits. Animal viruses are capable of fusing with prokaryotic cells that may facilitate gene transfer between distant microbial taxa [294]. Influenza virus hemagglutinin A sequences have been located in the p37 protein of *Mycoplasma hyorhinis*, and this protein increases tumor cell invasiveness [295]. The exchange of genes among various microorganisms [296] leads to the development of antibiotic resistance. Gene uptake also occurs by phagocytosis of apoptotic bodies [297,298] while High Mobility Group (HMG) proteins, commonly associated with human DNA, may facilitate this process in bacteria [299].

When antigens from pathogens mimic self-antigens in the process of molecular mimicry, cross-reactive T cells may be generated. The study on breaking T cell tolerance with co-administered foreign and self-cytochrome c is a sobering reminder of just how easy is to induce autoimmunity. However, evidence also demonstrates that a low level of autoimmunity is normal and necessary to mount an effective immune response to infections. Clinical autoimmunity may develop if a continuing high-dose antigenic stimulus persists, as in cases of chronic infection. In addition, there is also evidence that autoimmunity can lead to proliferative disorders.

As discussed, normal tissue development requires the elimination of dangerous and abnormal cells, and autoimmune T cells belong into this category. With the completion of the immune response, the evolutionarily conserved mechanism of apoptosis eliminates effector T cells, leading to immune response termination and tolerance development. However, defective apoptosis can lead to autoimmunity and cancer.

We propose that an aberration in the apoptosis process leads to formation of the cancer stem cell from autoreactive T cells. In support of this observation, *Helicobacter*-induced gastric epithelial carcinoma was found to originate from bone marrow-derived cells [300]. This is direct proof of cancer that is not arising from mutated epithelial cells. Also, the cytotoxic T lymphocyte-associated antigen-4 (CTLA-4), a regulator of the effector function of T cells, is expressed in various leukemias and solid tumors [304]. This suggests a link between CTLs, hematopoietic neoplasias and solid tumors.

Further evidence: the common acute lymphoblastic leukemia antigen was detected on glioma [301] and melanoma [302] cell lines. The melanoma-associated PRAME antigen is expressed both in leukemias and some solid tumors [303]. The majority of leukemia and lymphoma cells test positive for the leukocyte common antigen (CD45) [305]. Seminoma [306], rhabdomyosarcoma [307] and some metastatic undifferentiated and neuroendocrine carcinomas [308] have also been found to express CD45. The myeloid antigen Leu-7, typically expressed on natural killer (NK) cells and T cell subsets, was detected on small cell lung carcinoma [309,310] and a variety of other solid tumors including astrocytoma, neuroblastoma, retinoblastoma, carcinoid tumors, etc. [311]. Neoplastic cells of Hodgkin's disease expressing Leu-7 may be related to NK cells or T cells rather than B cells [312]. We propose that the unexpected presence of some T cell markers on cancer cells may provide an insight into their origins. In addition, the observation that cancer stem cells embedded in an environment of normal host tissue can undergo a differentiation process (during which surface markers of lymphoid origin disappear) explains the absence of leukocyte-derived surface antigens in some solid tumors.

In benign colonic adenomatous polyps and synchronous adenocarcinoma, comparable and very large numbers of genomic alterations (>10,000 events per cell) were found [313], demonstrating massive genomic damage characteristic of apoptosis as opposed to sequential mutations. In addition, this demonstrates that genomic instability precedes the development of a malignant state, indicating that malignancy is an effect rather than the cause of genetic abnormalities in cancer cells. It is therefore reasonable to conclude that there is no fundamental difference between benign and malignant tumors, and that possibly just a small difference in the disregulation of proliferative controls leads to a malignant phenotype.

We further propose that the resultant cancer stem cell still preserves some functions of an effector T cell, such as homing in to sites of inflammation such as the inflamed bronchi of a cigarette smoker, the damaged liver of an alcohol abuser, an *H. pylori*-infected gastric mucosa, an HPV-infected uterine cervix, an inflamed colon, etc. The cancer cell retains some capabilities of an effector T cell to secrete inflammatory cytokines (even if in an aberrant, constitutive fashion), thereby distorting local immune responses, disabling cytotoxic T cells and diminishing apoptosis in its environment.

Like normal activated inflammatory cells, cancer cells activate the coagulation system, leading to the formation of the tumor stroma in which tumor cells proliferate. Dvorak in his paper entitled "Tumors: wounds that do not heal" [91] succinctly described similarities between the formation of the temporary stroma of a healing wound and tumor stroma development. While the cancer cell continues to act as if it participated in a wound healing process, it actually enlarges the wound stroma due to its constitutive secretion of tissue factor, inflammatory cytokines and other growth factors which also provide stimuli for the propagation of the malignant cells. This leads to an ever-continuing cycle of tumor growth.

Every human cell has the ability to repair itself, and cancer cells retain some of this capacity [314]. As cancer stem cells exhibit plasticity similar to normal stem cells, we propose that a cell-to-cell communication between cancer stem cells and surrounding host tissues allows tumor cells to develop varying degrees of differentiated phenotypes resembling cells of normal differentiated tissues. This in turn leads to the emergence of various tumor types and creates the illusion of a great multitudes of cancers.

It has been long known that cancer cells, besides growing inside tumors, also circulate in the blood [315-317]. This is easy to rationalize if cancer cells are indeed damaged autoreactive T cells, and also provides an explanation for metastasis formation. Cancer cells interact with neutrophils, macrophages and platelets that lead to the formation of micrometastases that can remain in the blood for a long time [318]. These aggregates persist even after adjuvant chemotherapy, although in reduced numbers. Larger cell clumps are more effective in promoting metastases than smaller ones [319]. With the progression of inflammation in cancer patients, the circulating micrometastases find new sites of proliferation that lead to the formation of metastases.

Current cancer therapies are tumor-centric, as tumors are equated with cancerous disease. Main therapeutic modalities include the surgical removal of tumors as well as radiation and chemotherapies. All of these contribute to the hypercoagulable state and risk of thromboembolism, which have a significant negative impact on the morbidity and mortality of cancer patients. If tumor cells did originate from T cells, any therapeutic approach targeting tumor cells will likely diminish T cell function. Cytotoxic antineoplastic therapy represents an extreme situation in this regard, resulting in the deletion of even resting T cells, the reconstitution of which takes several months [10]. This makes the combination of chemotherapy and immunotherapy an unrealistic proposition.

If cancer cells indeed originate from damaged autoreactive T cells, our current views on cancer immunotherapy need to be revised. The immune system was not made to attack itself, and this is supported by the unresponsiveness of the cellular immune system to cancer even if tumor cells are antigenic. When we attempt to induce an immune response against tumors, we run the risk of developing autoimmune disease [320] and ultimately, secondary malignancies.

The suppression of the immune system by chemotherapeutic agents and radiation encourages the propagation of microbial and parasitic infections already present in cancer patients. However, pathogenic microorganisms are intimately involved as co-etiological agents in the development of various malignancies via molecular mimicry-induced autoimmunity, and maintain a cytokine milieu that favors proliferation as opposed to apoptosis. Current immunosuppressive cancer therapies establish the conditions for disease recurrence as well as the emergence of new primary tumors, which is in fact, a common experience. Also, the cancer patient's system appears to retain a "memory" of the disease as the risk of developing another cancer is higher than those who have never had the disease. This memory could be attributed to autoimmune memory T cells, reactivated by recurrent infections which become cancerous later on as a consequence of defective apoptosis.

The eradication of pathogens could have a favorable effect on the course of malignant diseases, as demonstrated by therapies of HCV [150], *H. pylori *[154], and *Chlamydia psittaci *infections [156]. Mycoplasmas are difficult to eradicate and require high-dose, long-term antibiotic therapies, but even after that the pathogens are found to persist [321]. There are no therapies for many viral infections at this time. With our new understanding of the mechanism of TLR signaling, opportunities have opened for overcoming these types of pathogens. Very recently, a therapeutic oral mycoplasma vaccine was described [322], the principle of which could be utilized for the therapy of other intracellular infections.

If defective apoptosis of autoreactive T cells leads to the emergence of the cancer stem cell, our research must focus on the physiological events associated with apoptosis. Any therapeutic approach downstream from this step is merely symptomatic, and offers little hope of defeating cancer. A century of accumulated evidence on the use of immunosuppressive cancer therapies supports this observation.

It was demonstrated that the exterior mucopolysaccharide cell surface coat on cancer cells protects them from apoptosis [52,53]. Kovacs has explored this understanding to the greatest degree by synthesizing unsaturated aminolipids capable of displacing the cell coat on tumor cells [45]. Administration of these compounds led to the apoptotic death of a variety of tumor cells *in vitro *and *in vivo *[45]. Normal lymphocytes are less sensitive to the apoptotic effects of a fatty acid mixture than leukemic cells, although they do show some sensitivity [323]. This observation may explain why the continuing administration of synthetic unsaturated aminolipids led to a diminishing efficacy of the therapy [324], as normal lymphocytes are also surrounded by an exterior cell surface layer coat essential for their functions.

Endocrine hormonal signaling also affects apoptosis. Corticosteroids facilitate the apoptosis of lymphocytes and exert an immunosuppressive effect when the organism is subject to prolonged stress. Stress also down-regulates the digestive functions of the gut, including those of the stomach and pancreas. This in turn suppresses the uptake of critical nutrients that are essential for genomic stability [14]. It was reported that breast cancer patients as a group exhibit a depressed thyroid function [14], suggesting an etiological role for thyroid deficiency in neoplasia. Thyroid function is profoundly affected by the iodine supply, and thyroid, breast and gastric cancers have been linked to iodine deficiency [14]. Previously we have pointed out that critical nutrient deficiencies mimic the effects of chemical or radiation damage to DNA, and suggested that the correction of these deficiencies could reverse the progression of malignant proliferation [14].

In the past century, insufficient attention was paid to the role of dietary factors in the development and progression of malignant diseases. No Recommended Daily Allowances (RDAs) are available for a number of essential nutrients, and where available, the RDA is of questionable value. Iodine, a vital micronutrient, is an example: the current WHO recommendation for iodine is 0.15 mg/day. However, some Japanese consume as much as 50–80 mg of iodine/day through their seaweed rich diet [325] and exhibit significantly lower rates of the major cancer types than seen in the Western world [14]. In addition, iodine supplementation clinical trials have demonstrated that an iodine intake vastly exceeding the RDA (more than 6,000 times higher) was both safe and clinically useful [326,327]. This could not possibly be the case if the RDA for iodine had been correctly determined. Similar clinical observations were made for high-dose administration of folate and vitamin B_12 _[328,329] as well as vitamin C [330]. These findings question the accuracy of dietary RDAs, and suggest that current regulatory initiatives aimed at restricting the active ingredient contents in vitamin supplements are based on an erroneous scientific rationale.

It is also important to recognize that vitamin and mineral levels have significantly declined over the past 60 years in our food supply [reviewed in [331]] possibly due to intensive agricultural production methods and industrial food processing. Experience teaches us that in the Western world, despite an abundance of food, people have difficulties in meeting their nutritional needs, demonstrated by now-rampant obesity as well as the historically proven explosion of degenerative diseases including cardiovascular diseases, diabetes and cancer. This suggests that we are still far from understanding the dietary needs of the human organism.

It is known that diabetics develop malignancies at a higher frequency than the population average [332,333], which implicates pancreas dysfunction in the etiology of cancer. Besides secreting digestive enzymes, the pancreas is also a source of hormonal regulators. We hypothesize that a combined effect of adrenal, thyroid and pancreas dysfunction may predispose patients for neoplasia in a process promoted by dietary deficiencies as well as lifestyle factors including prolonged stress, poor hygiene, smoking, alcoholism and drug abuse, all of which are known to subvert immunity. It appears that we need to make the most important scientific discoveries in the simplest things, i.e., how to conduct our lives in a manner optimal for well-being. Therefore, the main operative principle of health care should be prevention.

To finally defeat cancer, our research need to focus on the identification of those endocrine-signaling mechanisms that enable CTLs to complete their mission of apoptotic elimination of autoreactive T cells. We must abandon our focus on the tumor cell as far as the development of cancer therapeutics are concerned, as the destruction of cancer itself negatively impacts the immune system, thereby reactivating the vicious circle of infection, autoimmunity and malignancy that ultimately dooms cancer patients. By redirecting our focus toward physiological events preceding the formation of the cancer stem cell, we will be able to overcome this scourge that has haunted humanity since time immemorial. A systemic approach described in a previous paper [14] offers an alternative to current cancer therapies that works with the immune system, and which helps to re-establish homeostatic balance in the human body.
